# Relationship between metabolic indices and milk yield in Saanen goats exposed to heat stress in semi-tropical climates

**DOI:** 10.1007/s11250-024-04097-6

**Published:** 2024-08-12

**Authors:** Fatma Akkaya, Zafer Mecitoğlu, Sezgin Şentürk, Nedim Koşum, Sevim Kasap, Çiğdem Takma, Turgay Taskin, Murat Yalçin, Çağrı Kandemir

**Affiliations:** 1 Faculty of Agriculture, Department of Animal Science, Ege University, İzmir, 35100 Turkey; 2 Faculty of Veterinary Medicine, Department of Internal Medicine, Bursa Uludağ University, Bursa, Türkiye; 3 Faculty of Veterinary Medicine, Department of Physiology, Bursa Uludağ University, Bursa, Türkiye

**Keywords:** Heat stress, Milk yield, Warming, NEFA

## Abstract

This study aimed to determine the effects of heat stress on 30 Saanen goats of different ages (young, middle-aged, and old). The average temperature and humidity values during the trial were 27.82 °C and 40.65%, respectively. Statistically significant differences in pulse rate (PR), respiratory rate (SS), and temperature humidity index (THI) were found between months (*P* < 0.05). Beta-hydroxybutyric acid (BHBA) values were found to be statistically significant in the young and middle-aged group (*P* < 0.05), with the highest in the middle-aged group obtained in June (0.65 mmol/L). Non-esterified fatty acids (NEFA) and urea (BUN) were significantly different (*P* < 0.05) in all age groups, whereas creatinine values showed no significant differences between groups. Significant positive relationships were found between body weight (BW) and body condition score (BCS), as well as pulse rate (PR) and daily average daily milk yield (DAMY) (*P* < 0.01). A positive correlation was also found between the respiratory rate (SS) and pulse rate (*P* < 0.01). As a result, although attempts have been made to prevent the decrease in productivity with applications for animals under heat stress, the optimal strategy may be to address the source of the problem. Issues that cause heat accumulation in the body should be identified and necessary arrangements should be made in the shelter to distribute heat to ensure that animals are less affected. Shaded areas should be provided in cases of stress caused by heat. An appropriate structural arrangement for temperature, humidification, and ventilation systems, as well as the provision of abundant fresh drinking water, would also be beneficial.

## Highlights

Decreased milk yield is related to thyroid hormones which speed up metabolic processes by increasing enzyme activity.

A significant decrease in milk yield was observed in both the young and old groups in July and August, when heat stress was most prevalent.

The middle-aged group was least affected by heat stress in terms of milk yield.

Changes in creatinine levels were insignificant, only determining a significant decrease in urea levels.

Despite decreased feed intake, no rise in plasma NEFA was observed.

## Introduction

In recent years, climate change has increased the awareness of the effects of heat stress on animals (Alsharif 2022). Saanen goats are strongly affected by heat stress due to their robust milk metabolism (Kandemir et al. 2013; Yilmaz et al. 2018a; Lima et al. 2022). Recent studies have shown that heat stress has a significant effect on metabolic profiles, including energy, liver, and hormonal metabolism, which lowers milk production (Hooper et al. 2018; El-Gindy et al. 2022; Choi et al. 2021; Lima et al. 2022). To circumvent this, some scientists have focused on minimizing the heat stress experienced by animals by improving the environment in which they are maintained (Saipin et al. 2020; Choi et al. 2021; Salem et al. 2022; Hanafi et al. 2022).

In hot, dry regions where the ambient temperature may surpass the upper critical threshold of animals, environmental and climatic stressors both play a significant role in animal production. According to previous reports, goats are less susceptible to stress factors than other domesticated ruminants (Khalifa et al. 2005). Goats can adjust to hot and dry conditions because of their ability to conserve water, basal metabolic rate, high respiratory rate, high skin temperature, and constant cardiac output. However, heat stress may still impair the rumen fermentation and growth efficiency of goats (Xue et al. 2022). Indeed, research has demonstrated that exposure to heat stress reduces the number of cellulolytic bacteria and increases the abundance of bacteria that break down starch (Xue et al. 2022).

In Turkey, many residents in both urban and rural areas raise goats to address their milk needs (Daskıran et al. 2018). This study was designed to compare how heat stress affects the metabolism of dairy goats of different age groups during the summer season in Turkey, in order to devise strategies to help mitigate the effect of global warming on livestock.

## Materials and methods

### Animals

This study was conducted on 30 Saanen goats raised at the Farm Animal Production, Research, and Application Centre of the Faculty of Agriculture, Ege University. The goats used in this study were subjected to a general health examination before the study, and received annual preventive immunizations at regular intervals. The goats were divided into three age groups: young (average age: 1.5 y), middle-aged (average age: 3.5 y), and older (average age: 5.5 years old). The animals did not become pregnant during the experiments. All experiments were approved by the Ege University Local Animal Experiments Ethics Committee (2020-082).

### Location, feeding, and management

The experimental study continued for 4 months (May–August), during which the goats were in the mid-lactation period. Izmir, where the study was conducted, is a city located on the seaside of the Aegean region of Turkey and thus has a Mediterranean climate. June, July, and August are considered the summer months. In the Aegean region, beginning in May, animals remain at a high temperature for four to five months. Therefore, these months were chosen as the optimal time to study animal heat discomfort. The daily temperature, relative humidity, and humidity index were recorded monthly. All animals were provided with 0.8 kg of pelleted feed, 2.0 kg of corn silage, 0.2 kg of wheat straw, and 0.6 kg of dry alfalfa grass daily as feed, provided using a feed-vending machine equipped with a radiofrequency identification system. The daily average milk yield (DAMY) of the goats was determined, and additional milk feed requirements were identified.

### Sampling and data

Throughout the experiment, DAMY data were gathered from the herd management system. Body weight (BW) and body condition score (BCS) were measured twice a month. The heart rate of the goats was also measured between the left 3–6 costa, and the respiratory rate (RR) was calculated based on the flank’s motion. A digital thermometer (KERBL^®^, Ankara, Turkey) was used to measure the rectal temperature of the goats. The pulse rate (PR) (beats/min), respiratory rate (RR) (beats/min), and rectal temperature (RT) (0 C) of the goats were measured twice daily (08:00–09:00 a.m. and 2:00–3:00 p.m.) throughout the study period. Blood samples (2 ml) were collected from the jugular vein in vacuum tubes without anticoagulants twice a month. During processing, blood samples were first allowed to clot, after which they were centrifuged at 3000 rpm for 10 min at 4 0 C to obtain serum. The serum samples were the collected and stored at − 20 °C until analysis of biochemical hormonal parameters. Biochemical tests were conducted using Randox test kits (Randox Laboratories Ltd., Crumlin, UK). The enzyme aspartate aminotransferase (AST) was measured using the UV (Modified IFCC) technique (catalogue number: AS3804). The colorimetric method was applied to measure the levels of total protein (TP) (catalogue number: TP3869), beta-hydroxybutyric acid (BHBA) (catalogue number: RB1007), non-esterified fatty acids (NEFA) (catalogue number: FA115), gamma-glutamyl transferase (GGT) (catalogue number: GT8320), and lactate (LACT) (catalogue number: LC3980). Biuret and enzymatic kinetic procedures were employed to assess urea (BUN) (catalogue number: UR3825) and creatinine (CREA) (catalogue number: CR3814).

### Statistical analysis

Any differences in metabolic and physiological measurements for goats of the same age according to month were analyzed using GLM univariate analysis. Tukey’s multiple comparison test was applied to perform multiple comparisons of means (SAS 1999).

## Results

**Fig. 1:**
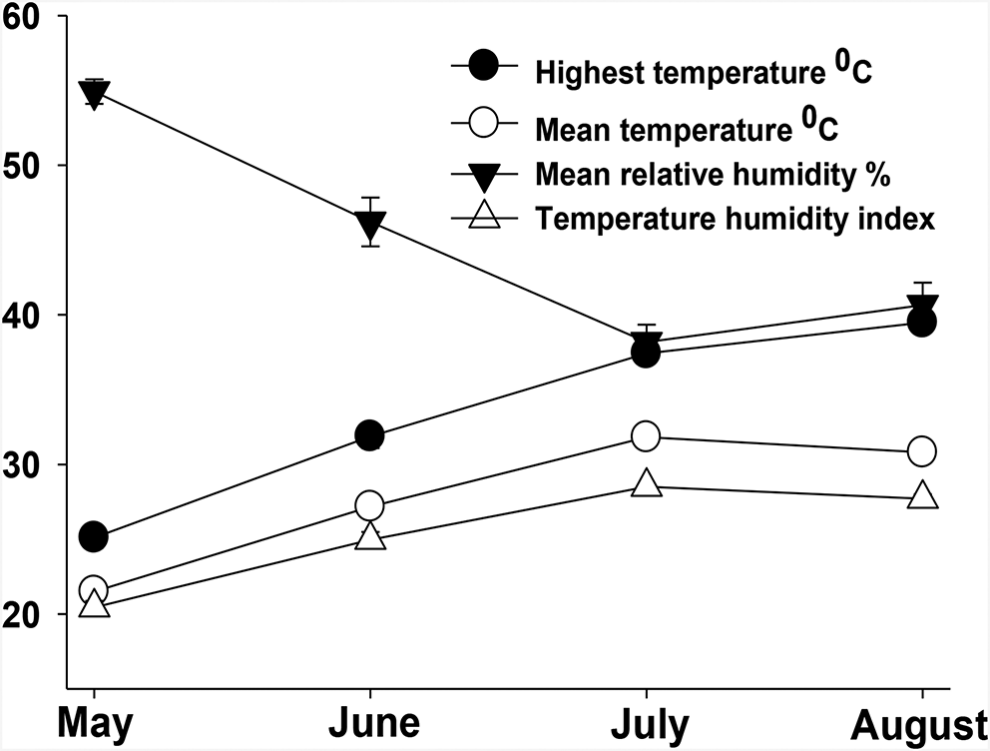
The temperature, relative humidity, and humidity index measurements for each month of the experiment. Data shows mean ± SEM of the monthly mean of daily measurements

Descriptive statistics of the climatic data according to month are shown in Fig. 1. According to our findings, while the highest temperature (HT, °C) in May was 25.11 °C, this value increased to 39.48 °C in August, yielding a temperature rise of more than 14 °C. The average temperatures in May, June, July, and August were 21.50 °C, 27.18 °C, 31.82 °C, and 30.81 °C respectively. During the entire study period, the mean temperature overall was 27.82 °C, and the average humidity value was 40.65%. The difference between the months in terms of HT (°C), AT (°C), AH (%), and the temperature humidity index (THI) was found to be statistically significant (*P* < 0.05).

BW did not affect heat stress in goats of any age group, whereas BCS was found to have an impact on the young and middle-aged groups (*P* < 0.05). Only the middle-aged group showed a substantial impact of RR, while DAMY, RT, and PR were statistically significant in all other age groups (*P* < 0.05). In all groups, DAMY decreased significantly in the months with the highest heat stress, while RT, PR, and RR increased the most in August (Table 1).

**Table 1:** Heat stress’s physiological consequences on goats

	Young					Middle					Old				
	Ma	Ju	Jl	Au	*P*	Ma	Ju	Jl	Au	*P*	Ma	Ju	Jl	Au	*P*
BW	64,31	65,99	68,50	71,23	0,36	69,77	70,12	71,64	74,03	0,68	63,16	62,66	62,85	64,18	0,99
BCS	2,5^b^	2,5^b^	2,5^b^	2,75^a^	< 0,01	2,5^a^	2,5^a^	2,5^b^	2,63^a^	< 0,01	2,5	2,5	2,13	2,25	0,32
DAMY	2,69 ^a^	2,41 ^ab^	2,24 ^bc^	1,97 ^c^	< 0,01	2,69 ^a^	2,47 ^ab^	2,27 ^ab^	2,06 ^b^	0,02	2,40 ^a^	2,01 ^ab^	1,84 ^b^	1,60 ^b^	< 0,01
RT	38,90^a^	39,00^a^	39,15^ab^	39,30^b^	0,03	38,6^a^	38,90^ab^	38,90^b^	39,00^b^	0,03	38,50^a^	38,75^ab^	38,95^ab^	39,00^b^	< 0,01
RR	29,30	31,90	30,22	36,80	0,47	24,70^b^	27,60^b^	28,00^ab^	31,40^a^	< 0,01	24,10^a^	28,30^ac^	29,30^bc^	32,80^b^	0,62
PR	107,00^a^	111,50^ab^	117,00^ab^	121,50^b^	< 0,01	98,00^a^	108,00^ab^	116,00^b^	121,00^b^	< 0,01	79,00^a^	89,00^ab^	98,00^b^	104,00^b^	< 0,01

a.b.c: The distinction between different letters on the same line is statistically significant; *:*P* < 0.05, **:*P* < 0.01

Ma: May; Ju: June; JL: July; Au: August; BW(kg): Bodyweight; BCS: Body condition score; DAMY(kg): Daily Average Milk Yield; RT (oC): Rectal temperature; RR(beat/min): Respiratory rate; PR(beat/min): Pulse rate; *P* < 0.05

The young group was the most affected by heat stress in terms of metabolic parameters. Indeed, all parameters except CREA were statistically significant in the young group (*P* < 0.05). Although CREA and LACT were not significant (*p* > 0.05) in the middle-aged group, all other parameters were statistically significant (*P* < 0.05). In the older group, only the TP, NEFA, and BUN values were significantly different (*P* < 0.05) (Table 2). Overall, a significant positive correlation was found between BW and BCS, as well as PR, and DAMY (*P* < 0.01) (Table 3). While a positive and significant relationship was found between RR and PR (*P* < 0.01), a significant negative relationship was found between RT, BHBA, and DAMY (*P* < 0.05). In contrast, the relationship between RR and BHBA level was negative and significant (*P* < 0.01). Conversely, the relationship between BUN and CREA, LACT, and DAMY were positive and significant (*P* < 0.01) (*P* < 0.05), and a positive and significant relationship was also observed between the CREA and LACT (*P* < 0.05).

**Table 2:** Related metabolic changes on by heat stress in goats

	Young					Middle					Old				
	Ma	Ju	Jl	Au	*P*	Ma	Ju	Jl	Au	*P*	Ma	Ju	Jl	Au	*P*
BHBA	0,49^bc^	0,61^a^	0,53^b^	0,38^c^	< 0,01	0,49^b^	0,65^a^	0,53^ab^	0,46^b^	0,01	0,34	0,46	0,40	0,28	0,17
TP	5,46^a^	4,74^ab^	4,35^ab^	3,54^b^	< 0,01	5,15^a^	4,86^a^	4,97^a^	3,38^b^	< 0,01	5,58^a^	5,55^a^	5,32^ab^	4,13^b^	0,02
AST	123,9^a^	109,7^a^	104,7^a^	70,5^b^	< 0,01	119,0^a^	113,9^a^	109,7^a^	75,5^b^	< 0,01	130,9	127,2	120,1	93,6	0,07
GGT	45,7^a^	37,1^ab^	40,4^ab^	30,5^b^	0,03	39,9 ^a^	34,8 ^a^	39,1 ^ab^	27,7 ^b^	< 0,01	41,1	38,8	36,6	30,1	0,1
NEFA	0,10^a^	0,12^a^	0,05^b^	0,04^b^	< 0,01	0,084	0,085	0,041	0,021	< 0,01	0,07^a^	0,08^a^	0,04^ab^	0,04^b^	0,04
BUN	27,32^a^	23,85^ab^	27,64^a^	19,20^b^	< 0,01	23,87^ab^	19,67^bc^	25,62^a^	16,77^c^	< 0,01	24,14^a^	20,44^ab^	24,69^a^	14,93^b^	< 0,01
CREA	0,629	0,644	0,651	0,584	0,40	0,610	0,617	0,627	0,585	0,76	0,627	0,651	0,657	0,61	0,72
LACT	14,70^a^	14,65^a^	10,66^ab^	8,07^b^	0,01	12,70	13,78	13,13	10,31	0,17	14,52	13,53	17,90	12,80	0,50

a.b.c: The distinction between different letters on the same line is statistically significant; *:*P* < 0.05, **:*P* < 0.01

Ma: May; Ju: June; JL: July; Au: August; BHBA(mmol/L): Beta-hydroxybutyric acid; NEFA(mmol/L): Non-esterified fatty acid; AST(U/L): Aspartate aminotransferase; GGT(U/L): Gamaglutamyltransferase; TP(g/dl): Total protein; BUN(mg/dL): Urea; CREA(mg/dL): Creatinine, LACT(mg/dL): Lactate; *P* < 0.05

**Table 3:** Phenotypic correlations between physiological and biochemical parameters in goats

	BBCS	RRT	RRR	PPR	BBHBA	TTP	AAST	GGGT	NNEFA	BBUN	CCREA	LLACT	DDAMY
*BBW*	,607^**^	-,021	,078	,239^**^	-,127	,094	-,101	-,141	-,035	-,101	,153	-,083	,311^**^
*BBCS*		,062	,092	,286^**^	-,053	,250^**^	,039	-,008	,104	,021	,190^*^	,036	,309^**^
*RRT*			,327^**^	,541^**^	-,125	,165	,137	,091	-,174	-,041	-,042	,144	**-**,215^*^
*RRR*				,519^**^	**-**,300^**^	-,096	-,098	-,171	-,147	-,110	-,178	-,067	-,095
*PPR*					**-**,**2**17^*^	,025	-,037	-,087	-,092	-,097	-,066	,047	-,079
*BBHBA*						,164	,125	,387^**^	,147	,405^**^	,084	,090	,235^*^
*TTP*							,574^**^	,652^**^	,355^**^	,317^**^	,604^**^	,351^**^	,051
*AAST*								,376^**^	,267^**^	,338^**^	,341^**^	,366^**^	-,031
*GGGT*									,250^**^	,402^**^	,373^**^	,190^*^	,156
*NNEFA*										,070	,158	,122	,100
*BBUN*											,316^**^	,214^*^	,249^**^
*CCREA*												,213^*^	,123
*LLACT*													,011

**: Correlation is significant at the 0.01 level (2-tailed). *: Correlation is significant at the 0.05 level (2-tailed)

BW: Bodyweight; BCS: Body condition score; DAMY: Daily average milk yield; RT: Rectal temperature; RR: Respiratory rate; PR: Pulse rate; BHBA: Beta-hydroxybutyric acid; NEFA: Non-esterified fatty acid; AST: Aspartate aminotransferase; GGT: Gamma glutamyl transferase; TP: Total protein; BUN: Urea; CREA: Creatinine, LACT: Lactate

## Discussion

Overall, it is necessary to develop sustainable management systems that consider the effects of climate change in the livestock industry. Conserving and spreading indigenous goat breeds that are climate-robust is one of several tactics that can be used to fight the harmful consequences of climate change. Goats are one of the livestock species with the highest climate tolerance across all continents. Environmental stress can be quantified by measuring the THI and correlating it with physiological, biochemical, or behavioral responses.

Many studies have shown that physiological shifts in PR, RR, and RT are the earliest signs of heat stress (Yadav et al. 2015; Kumar et al. 2020; Srivastava et al. 2021). In regards to rater (Gaughan et al. 2000; Phulia et al. 2010; Yadav et al. 2015; Kumar et al. 2018), goats increase their RR when exposed to heat to achieve homeothermy by respiratory heat loss, thus avoiding heat stress (Berbigier and Cabello 1990). The findings of a previous study revealed that at THIs of 71.78 and 79.48, respectively, PR was the first physiological parameter to change in both the Jamunapari and Barbari goat breeds, while goats native to India’s semi-arid region responded to environmental heat stress by increasing PR (Srivastava et al. 2021). Numerous studies have found that, as the PR increases, the muscles controlling the RR become more active, which functions to further increase the RR (Gupta and Mondal 2021). These data further demonstrate that the first sign of heat stress is an increase in PR. Studies on dairy cattle conducted by Jackson and Cockcroft (2008), Zimbelman et al. (2011), Dalcin et al. (2016), and Pinto et al. (2019) have all shown that the key THI for PR falls in the range between 68 and 73. Under heat stress conditions, a cascade of biochemical changes occurs in the body. For example, the body’s energy metabolism shifts toward maintenance, ultimately resulting in a loss of production when physical and physiological heat loss mechanisms fail to maintain homeothermy. In this study, in July and August, when heat stress was most prevalent, the goats in both the young and old groups showed a significant decrease in milk yield (Table 1). One previous study showed that a THI of 79 decreased milk output in Alpine goats, but did not affect Nubian goats (Brown et al. 1988). Furthermore, while Hamzaoui et al. (2013) reported a THI of 77 to be critical for Spanish Murciano-Granadina dairy goats, El-Tarabany et al. (2017) reported that a THI of 80 was critical for goats, and Sevi et al. (2001) suggested a critical THI of 80 in sheep. However, Hamzaoui et al. (2013) reported that goats suffered severe heat exhaustion at a THI of 85. Another study showed that Saanen goats showed reductions in daily milk yield of 3% and 13% at THIs of 81 and 89, respectively, (Sano et al. 1985). In the present study, the middle-aged group was found to be the least affected by heat stress in terms of milk yield, while the younger group was more adversely affected than the older group, resulting in lower milk yields. In addition, the RT values increased during the months that experienced the most severe heat stress. While maintaining a healthy body temperature is crucial for the production of milk in conditions such as mastitis, it is believed that febrile diseases takes longer to heal and/or have a worse prognosis because of the relative rise in temperature during the summer months. However, it could be stated that the decrease in milk yield is related to this because thyroid hormones accelerate metabolic processes by increasing enzyme activity. When an animal is subjected to heat stress, blood flow is concentrates in the periphery, water loss in the body increases, and movements slow down due to increased sweating and respiration rates (Contreras-Jodar et al. 2018; Bomfim et al. 2022). Owing to the disruption in circulation, the liver and udder tissues may not receive sufficient nutrients, which decreases milk production.

The values of all blood metabolites and enzymatic activities in the current study were within the typical reference ranges (Bertoni et al. 2020). In this study, we observed significant differences in BHBA, TP, AST, GGT, NEFA, BUN, and LACT levels. The current study’s observation of the impact of THI on energy metabolism is consistent with previous findings in goats under heat stress (El-Tarabany et al. 207; Yilmaz et al. 2018b). These effects could be attributed to several factors, including (1) decreased energy intake as a result of decreased DMI, (2) increased thermoregulation costs, (3) nutrient partitioning, and (4) the negative impact of heat on gluconeogenesis as a result of the body’s endocrine adaptation to hot conditions (Sorianiet al. 2013). Despite the decreased feed intake, no increase in plasma NEFA was observed, which is consistent with the findings of other studies investigating animals under heat stress (Rhoads et al. 2009; Shwartz et al. 2009). The evaluation of hematological parameters is crucial for determining how animals react to various stressful situations, as they are reliable markers of an individual’s physiological health status (Attia 2016). To assess the general health of the animals, we assessed serum enzymatic activity. According to Duncan and Prasse (1994), heat stress decreases the plasma levels of the enzymes aspartate aminotransferase (AST) and alanine aminotransferase (ALT), albeit within normal physiological bounds. Regarding renal function outcomes, Abdel-Samee et al. (2008) found that under heat-stress conditions, the blood levels of urea and creatinine significantly decreased. Although our study showed that the changes in creatinine levels were insignificant, only a significant decrease in urea levels was observed. This may be explained by a decrease in ruminal Ammonia-N, which is offset by an increase in Urea-N absorption in the rumen, causing a decrease in blood urea levels, and an increase in urine nitrogen excretion. These findings were consistent with those reported by Fike et al. (2005). It has been shown that there were considerable variations in blood biochemical levels, which may be the result of an altered metabolism triggered by stress in the goats.

## Conclusion

When the ambient temperature exceeds the range of an animal’s thermoneutral zone, a combination of environmental conditions result in a condition known as heat stress. Feed intake, growth rate, and haemato-biochemical profile of goats were all negatively impacted by heat stress. During some periods of the trial, the dairy goats used in this study experienced mild to moderate heat stress. In addition to an increase in breathing rate and rectal temperature, the physiological markers of heat stress assessed in this study demonstrated that hot conditions affected hematological variables linked to energy, protein, and enzyme activity (Kandasami et al. 2023; Kaushik et al. 2023). As a result, although attempts have been made to prevent the decrease in productivity with applications for animals under heat stress, the optimal strategy would be to mitigate the source of the problem. Issues that cause heat accumulation in the body should be identified, and necessary arrangements should be made in animal shelters to distribute heat and ensure that the animals are less affected by high temperatures. Shaded areas should be provided in case of stress caused by heat. An appropriate structural arrangement for temperature, humidification, and ventilation systems along with the provision of plenty of fresh drinking water would also be beneficial.

## Data Availability

The data that support the findings of this study are available upon reasonable request from the corresponding author.
